# Neural Decoders Using Reinforcement Learning in Brain Machine Interfaces: A Technical Review

**DOI:** 10.3389/fnsys.2022.836778

**Published:** 2022-08-26

**Authors:** Benton Girdler, William Caldbeck, Jihye Bae

**Affiliations:** Department of Electrical and Computer Engineering, University of Kentucky, Lexington, KY, United States

**Keywords:** reinforcement learning (RL), neural decoder, brain machine interface (BMI), neural interface, value function approximation, policy optimization

## Abstract

Creating flexible and robust brain machine interfaces (BMIs) is currently a popular topic of research that has been explored for decades in medicine, engineering, commercial, and machine-learning communities. In particular, the use of techniques using reinforcement learning (RL) has demonstrated impressive results but is under-represented in the BMI community. To shine more light on this promising relationship, this article aims to provide an exhaustive review of RL’s applications to BMIs. Our primary focus in this review is to provide a technical summary of various algorithms used in RL-based BMIs to decode neural intention, without emphasizing preprocessing techniques on the neural signals and reward modeling for RL. We first organize the literature based on the type of RL methods used for neural decoding, and then each algorithm’s learning strategy is explained along with its application in BMIs. A comparative analysis highlighting the similarities and uniqueness among neural decoders is provided. Finally, we end this review with a discussion about the current stage of RLBMIs including their limitations and promising directions for future research.

## Introduction

Reinforcement learning (RL) has been actively considered in robotics (Kober et al., 2013) to accomplish industrial automation (Meyes et al., 2017; Stricker et al., 2018) and humanoid robot behaviors (Peters et al., 2003; Navarro-Guerrero et al., 2012) and in business management to guide decision making (Huang et al., 2011; García et al., 2012), pricing strategies (Kim et al., 2016; Krasheninnikova et al., 2019), and stock price prediction (Jae Won, 2001; Wu et al., 2020). The unique mechanism of RL tries to mimic the human learning process that acquires knowledge based on experience in a trial-and-error manner. That is, in RL, the learning system not only observes but also interacts with the environment to collect information to accomplish the goal of a task. This unique mechanism provides a general framework for a system to adapt to novel environments.

Due to its advantages, flexibility for adaptation, and successful performances in difficult domains such as those mentioned above (robotics and business management), RL has been incorporated in a wide variety of domains, including autonomous driving (Zhao et al., 2020), natural language processing (Sharma and Kaushik, 2017), and search engines (Hu et al., 2018). In addition, RL has started to get more attention in medical applications (Gottesman et al., 2019; Coronato et al., 2020), including clinical decision support (Liu et al., 2020) and brain machine interfaces (BMIs).

Research in BMIs is a multidisciplinary effort that involves fields such as neurophysiology and engineering. Developments in this area have a wide range of applications, especially for people with neuromuscular disabilities, for whom BMIs may become a significant aid. Neural decoding of neural signals is one of the main tasks that need to be executed by the BMI.

In a neural decoder, various signal-processing and machine-learning techniques that find a map from neural signals to control commands for external devices have been explored (Kao et al., 2014; Xu et al., 2019). Conventional signal-processing techniques, including the Kalman filter (Kim et al., 2008), Kalman filter variations (Li et al., 2009; Gilja et al., 2012; Pandarinath et al., 2017), and Wiener filter (Salinas and Abbott, 1994; Carmena et al., 2003; Hochberg et al., 2006), have shown successful performances in neural decoding. An impressive example describing closed-loop BMI cursor control experiments on humans with tetraplegia can be found in Kim et al. (2008), where an average error rate of 13.8% was reported for one subject using the Kalman filter, called velocity Kalman filter, to decode the subject’s intracortical neural signals into two-dimensional velocity vectors of the cursor, (v_*x*_,v_y_). In addition, a variant of the Kalman filter, called recalibrated feedback intention-trained Kalman filter, has been integrated with a hidden Markov model-based state classifier to control a computer cursor that types on a virtual keyboard. This closed-loop experiment was conducted by decoding intracortical neural signals from subjects with amyotrophic lateral sclerosis and spinal cord injury, and the neural decoder showed competitive performances on typing tasks (average typing rate of 28.1 correct characters per minute and bitrate of 2.4 bits per second) (Pandarinath et al., 2017).

Moreover, supervised learning algorithms, such as support vector machines (Hortal et al., 2015; Toderean and Chiuchisan, 2017; Skomrock et al., 2018) and artificial neural networks, particularly recurrent neural networks (Oliver and Gedeon, 2010; Sussillo et al., 2012), have been actively considered in BMIs for neural decoding. It has been shown that a recurrent neural network can outperform the velocity Kalman filter in a closed-loop intracortical BMI (Sussillo et al., 2012). In addition, the closed-loop decoder adaptation strategy allows synergistic online adaptation for both user and neural decoder providing better interaction of the user with the environment through the BMIs and improved performance (Orsborn et al., 2011, 2012; Gilja et al., 2012; Shanechi et al., 2016; Brandman et al., 2018). Furthermore, following recent advances in deep-learning techniques, researchers have started investigating various deep-learning algorithms in BMIs (Mahmood et al., 2019; Mansoor et al., 2020).

Although these learning approaches have been applied to neural decoding in real-time control of BMIs, this is probably not the most appropriate methodology for paraplegic users because of the absence of ground truth. The basic mechanism of the above-mentioned signal processing and machine learning approaches is as follows: given a training set of neural signals and synchronized movements, the problem is posed as finding a mapping between these two signals, which can be solved by applying supervised learning techniques. That is, the kinematic variables of an external device are set as desired signals, and the system is trained to obtain the regression model. Unfortunately, the desired signal is determined by the experimenter, not by the user. In practice, since the user cannot move, the required information of the desired signal at each time instant to update the external device’s movement is missing. In addition, even if the desired signal is available, functionality is still limited to various task types or changing environments since frequent calibration (retraining) becomes necessary.

RL is one of the representative learning schemes, which provides a general framework for adapting a system to a novel environment inspired by how biological organisms interact with the environment and learn from experience. RL allows learning using only information from the environment, and thus there is no need for an explicit desired signal. Although RL does require a reward signal to guide the learning process, it is important to note that the reward can be obtained based on the user’s neural activity (Schultz et al., 1998; Marsh et al., 2015; An et al., 2019). These characteristics are well suited for the neural decoding task in BMI applications since BMIs need to have direct communication between the central nervous system and the computer that controls external devices such as a prosthetic arm for disabled individuals. Moreover, BMIs should be able to continuously adapt and adjust to subtle neural variations.

In this article, we focus on various RL methods that have been used in BMIs for neural decoding. Although preprocessing of the acquired neural data is an important step in BMIs, in this study, we do not place emphasis on the data preprocessing steps. In addition, interactive RL, which uses human guidance to optimize learning procedures, has been highlighted in BMIs (Cruz and Igarashi, 2020; Poole and Lee, 2022). The human feedback has been largely related to modeling rewards in RL. Modeling reward is another important step in RL, and there have been various attempts to model reward based on neural signals (Iturrate et al., 2010; Marsh et al., 2015; An et al., 2018; Shen et al., 2019). However, in this article, we focus on RL models used as a neural decoder in BMIs. Thus, studies solely based on modeling the rewards are out of the scope of this review.

To the best of our knowledge, this work is the first attempt to provide an exhaustive review of neural decoding algorithms applied to RLBMIs. In this article, we describe various RL methods that have been used in BMIs to adjust the parameters of the neural decoders and provide a summary of their advantages and limitations. It is expected that this review will not only serve as a reference guide for researchers already working in RL-based BMIs but also as an introductory tool to those that may be considering incorporating RL algorithms into their BMI work. The contributions of the authors include listing update rules and diagrams from different RL neural decoders with unified notation over different studies and providing a taxonomy for various neural decoders by categorizing their RL base model and type of function approximation algorithms. Experimental set up and details are also summarized along with reported neural decoder’s performances. This article is organized as follows: Section “Search Methodology” shows the methodology for the literature review process. Section “Background on Reinforcement Learning” provides the taxonomy and problem formulations in RL. Section “Reinforcement Learning Brain Machine Interfaces: Basic Mechanism” provides an overview of RLBMIs. Section “Reinforcement Learning in Brain Machine Interfaces: Neural Decoding Algorithms” reviews various types of neural decoders applied in RLBMIs. Section “Discussion” discusses future directions for research in RLBMIs.

## Search Methodology

**FIGURE 1:**
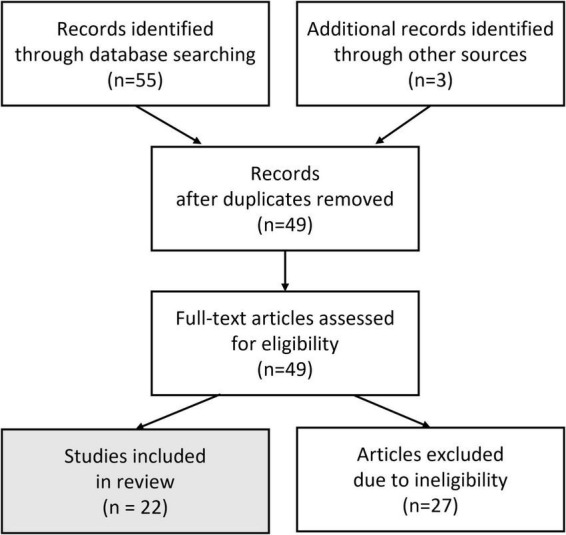
A review flow chart, by following the Preferred Reporting Items for Systematic reviews and Meta-Analyses (PRISMA) guidelines (Page et al., 2021).

We chose to search for relevant literature through the following databases: PubMed, JSTOR, Academic Search Complete, and Google Scholar. The phrases we employed were “Reinforcement Learning Brain Machine Interfaces” and “Error Related Potentials and Brain Machine Interfaces.” Once all seemingly relevant papers were gathered across the different databases based on their abstracts, replicates were removed, i.e., the same paper from different databases. From there, articles were removed after full-text analysis revealed they were not appropriate for our review, in the sense that the phrases used above were only superficially related to the paper (Figure 1).

In addition, Table 1 displays an itemized summary of the reviewed neural decoders in RLBMIs. The first column shows the main author and the publication year of the reported study. Neural decoder type is divided into three subcategories including RL base model, function approximator, and learning algorithm. Neural signal and subject types are listed in the subsequent columns, along with the number of subjects considered in the RLBMI experiments. The “Subject” column provides gender and specific species if available, when an animal study was conducted. The eighth column shows the type of task the subject conducted while the neural signal was acquired. “External device” shows the type of device that the subject was controlling. The tenth column shows the type of BMI experiments, if the subject was manually controlling the external device and pre-recorded neural signal was used with the neural decoder, it was listed as “Open,” and when the subjects’ neural signals were directly controlling the external device regardless of their behavior, it was marked as “Closed.” The highlighted performance was summarized under “Key reported performance.” The best reported performance is summarized in terms of success rate, for fair comparisons of all reported studies, and the data amount for evaluation is listed to provide an understanding of the learning speed. It should be noted that all provided information from the published studies has been summarized. However, there are fields that are missing some information as it was not available in the corresponding published studies.

**TABLE 1:** A summary of reviewed neural decoders in RLBMIs.

Author (year)	Neural decoder			Neural signal type	Subject		Task	External device	Closed or open loop	Key reported performance	
	RL base model	Function approximator	Learning algorithm		Type	No.				Best reported success rates	Data amount for evaluation
Huang et al. (2022)	Action-value function, Q	Linear function	P300 Linear Upper Confidence Bound (PLUCB)	EEG 32 channel	Healthy human	20	Symbol selections in a standard 6 × 6 matrix of symbols	2D screen	Open	Overall symbol accuracy: 80.4 12.8%	Two sessions (14 runs/session, 18 symbol selection/run, 6 sequence/symbol, and 12 flashes/sequence) 1st session: used a pseudo-detector to initialize the algorithm 2nd session: symbol detection
			Transferred P300 Linear Upper Confidence Bound (TPLUCB)							Overall symbol accuracy: 79.6 14%	
Bae et al. (2011)	Q-learning	Kernel expansion	Kernel Temporal Difference (KTD) (λ)	Intracortical M1 (185 units)	Female Bonnet Macaque	1	2-target center-out reaching task	2D Screen	Open	Around 100% after 2 epochs	43 trials/epoch Average over 50 Monte Carlo runs
Bae et al. (2015)	Q-learning	Kernel expansion	Kernel Temporal Difference (KTD) (λ)	Intracortical M1 (185 units)	Female Bonnet Macaque	1	2-target 1-step center-out reaching task	2D Screen	Open	2-target: 99% after 3 epochs	Average over 50 Monte Carlo runs 43 trials/epoch
							4-target 1-step center-out reaching task	2D Screen	Open	4-target: 99% after 5 epochs	Average over 50 Monte Carlo runs
							8-target 1-step center-out reaching task	2D Screen	Open	8-target: 98% after 6 epochs	Average over 50 Monte Carlo runs 8-target: 178 trials/epoch
							3 target 4-step center-out reaching task	2D Screen	Open	Above 60% after 1 epoch	
							4 target 2-step center-out reaching task	2D Screen	Open	Above 60% after 1 epoch	
				Intracortical M1 (14 units)	Marmoset Monkey	1	2-target reaching task	Robotic Arm	Closed	90% for Day 1	20 trials (10 trials each per target)
Bae et al. (2014)	Q-learning	Kernel expansion	Correntropy Kernel Temporal Differences (CKTD)	Intracortical M1 (49 units)	Female Bonnet Macaque	1	4-target center-out reaching task	2D Screen	Open	100% after 5 epochs	Average over 50 Monte Carlo runs 144 trials trials/epochs
Zhang et al. (2019a)	Q-learning	Convolutional neural networks (CNNs)	Dueling Deep Q Networks	EEG 14 Channels	Healthy Human	7	6 imagery action classification	N/A	Open	Average classification accuracy of 93.63%	34,560 samples per subject
DiGiovanna et al. (2007a)	Watkin’s Q(λ)	Feedforward neural network	Recursive Least Square	Intracortical	Rat	1	Go no-go task	Robotic Arm	Closed	93.7%	One session: 16 trials
DiGiovanna et al. (2007b)	Watkin’s Q(λ)	Feedforward neural network	Back-propagation	Intracortical M1 (25 units in left and 33 units in right hemisphere)	Rat	1	2-Target reaching task	Robotic Arm	Open	max observed 81.3% Avg.: 68.1 10.8%	10 sessions (16 trials/session)
DiGiovanna et al. (2009)	Watkin’s Q(λ)	Feedforward neural network	Back-propagation	Intracortical M1 (rat01: 16 units, rat02: 17 units, rat03: 29 units)	Male Sprague-Dawley Rat	3	2-target reaching task	Robotic Arm	Closed	Avg. performance: rat01: 68%, rat02: 74%, and rat93: 73%	Avg. 2.1 1.2 session (1 session/day) Avg. 141.6 41.3 trials
Sanchez et al. (2011)	Watkin’s Q(λ)	Feedforward neural network	Back-propagation	Intracortical M1 and PMd (total 190∼240 units)	Female Bonnet Macaque	1	8-target center-out reaching task	2D Screen	Open	Reached 100% after 18 epochs	43 trials/epoch
Tarigoppula et al. (2012)	Watkin’s Q(λ)	Feedforward neural network	Back-propagation	Simulated neurons	N/A	N/A	8-target center-out reaching task	2D Screen	Open	Over 95% with optimal Izhikevich-tuning depth	80 neurons
Wang Z. et al. (2015)	Attention-Gated Reinforcement Learning (AGREL)	Feedforward neural network	Attention-Gated Reinforcement Learning (AGREL)	Intracortical M1 (54 active channels)	Male Rhesus Macaque	1	4-target center-out reaching task	2D Screen	Open	Average target acquisition rate reached to 90.16%	Day 1, 2, 3, and 6 (40 min data/day) No repetition of the data considered.
Shen et al. (2020)	Attention-Gated Reinforcement Learning (AGREL)	Feedforward neural network	Attention-Gated Reinforcement Learning (AGREL)	Intracortical M1 (16 channel) and mPFC (16 channel)	Male Sprague Dawley	6	One level press task	Lever	Open	Average success rate of 87.5%	For six subjects over 300 training epochs multi day recordings
Zhang et al. (2020)	Attention-Gated Reinforcement Learning (AGREL)	Feedforward neural network	Transfer Learning and Mini-batch based Attention-Gated Reinforcement Learning (TMAGREL)	Intracortical M1, S1, and PPC (monkey01: total 480 neurons and Monkey02: total 396 neurons)	Male Rhesus Macaque	2	3-target reaching and grasping task	N/A	Open	Approximately 90% for both monkeys	Monkey01: 600 trails Monkey02: 300 trials
Li et al. (2016)	Attention-Gated Reinforcement Learning (AGREL)	Feedforward neural network	Maximum Correntropy based attention-gated reinforcement learning	Intracortical Premotor cortex (55 channels)	Rhesus Macaque	1	4-target obstacle avoidance task	2D Screen	Open	Average success rate 68.79%	Total 552 trials for 30 Monte Carlo runs
Wang et al. (2017)	Attention-Gated Reinforcement Learning (AGREL)	Kernel expansion	Quantized Attention-Gated Kernel Reinforcement Learning (QAGKRL)	Intracortical M1 (96 channels) and PMd (96 channels)	Male Rhesus Macaque	1	4-target obstacle avoidance task	2D Screen	Open	Average success rate of 80.83 10.3%	On one type of learning scenario Total 5000 trials
Zhang et al. (2018)	Attention-Gated Reinforcement Learning (AGREL)	Kernel expansion	Clustering based Kernel reinforcement learning	Four simulated neurons	N/A	N/A	4-target reaching task	2D Screen	Open	99.8 6.6%	20 Monte Carlo runs for 600 epochs
Zhang et al. (2019b)	Attention-Gated Reinforcement Learning (AGREL)	Kernel expansion	Clustering based Kernel reinforcement learning	Intracortical M1 (26 channels)	Male Macaque	1	4-target reaching task	Robotic Arm	Open	94.3 0.9%	20 Monte Carlo runs After 400 epochs 1000 data point/epoch
Zhang and Wang (2019)	Attention-Gated Reinforcement Learning (AGREL)	Kernel expansion	Clustering based Kernel reinforcement learning with a weight transfer	Three simulated neurons	N/A	N/A	Two level discriminative task	Lever	Open	Avg. approximately 95%	20 Monte Carlo runs 1000 trials for each task
Mahmoudi and Sanchez (2011)	Actor-Critic	Feedforward neural network	Back-propagation	Simulated Neurons	N/A	N/A	4-target reaching task (2D workspace)	Robotic Arm	Closed	Reached 98% after less than 200 trials per target	One session
				Intracortical M1 (20 units) and NAcc (23 units)	Male Sprague-Dawley Rat	1	2-target reaching task	Robotic Arm	Closed	reached 100% after 16 trials	One session: 40 trials
Pohlmeyer et al. (2012)	Actor-Critic	Feedforward neural network	Hebbian reinforcement learning	Intracortical M1 (21 signals) and NAcc (18 signals)	Marmoset Monkey (*Callithrix jacchus*)	1	2-target reaching task	Robotic Arm	Closed	Avg. 90% for the first 50 trials	Eight sessions (50∼60 trials/session)
Mahmoudi et al. (2013)	Actor-Critic	Feedforward neural network	Hebbian reinforcement learning	Simulated Neurons	N/A	N/A	2-target center-out reaching task	2D Screen	Closed	100% after 2 trials for 2 target tasks	One session
							4-target center-out reaching task	2D Screen	Closed	100% less than 50 additional trials for 4 target tasks	One session
				Intracortical M1 (20 signals)	Marmoset Monkey (Callithrix Jacchus)	2	Go no-go task	Robotic Arm	Open	Over 95% after 20 trials for both monkeys	Three sessions (1 session/day)
Pohlmeyer et al. (2014)	Actor-Critic	Feedforward neural network	Hebbian reinforcement learning	Intracortical M1 (monkey01: avg. 18.3 3.1 signals and monkey02: avg. 21.1 0.4 signals)	Marmoset Monkey (Callithrix Jacchus)	2	Go no-go task	Robotic Arm	Open	Avg. 94%: monkey01 Avg. 90%: monkey 02	1000 sessions: monkey01 200 sessions: monkey 02
									Closed	Avg. 93%: monkey01 Avg. 89%: monkey02	Four sessions (1 session/day)
Prins et al. (2014)	Actor-Critic	Feedforward neural network	Hebbian reinforcement learning	Intracortical M1 (20 signals)	Marmoset Monkey (Callithrix Jacchus)	1	Go no-go task	Robotic Arm	Open	From 77 to 83% when Critic accuracy is 90%	100 trials/session
Roset et al. (2014)	Actor-Critic	Feedforward neural network	Hebbian reinforcement learning	EEG Nine channels	Subject with Chronic Spinal Cord Injury	1	Hand grasp or open task	Functional Electrical Stimulation Device	Closed	Avg. around 65%	Four closed-loop session (1st session: 300 trials, 2nd and 3rd sessions: 450 trials, and 4th session: 300 trials)

*EEG, electroencephalogram; M1, primary motor cortex; mPFC, medial prefrontal cortex; NAcc, nucleus accumbens; PMd, primate dorsal premotor cortex; PPC, posterior parietal cortex; S1, somatosensory cortex. Note that in Neural Signal Type, when the input state includes both single and multi-unit activities, a term “signal” was used, as the authors used this term in their studies.*

## Background on Reinforcement Learning

In RL, a controller, called an agent, interacts with a system, called the environment, over time and modifies its behavior to improve performance. This performance is assessed in terms of cumulative rewards, which are assigned based on the task goal. The agent tries to adjust its behavior by taking actions that will increase the cumulative reward in the long run; these actions are directed toward the accomplishment of the task goal.

An RL framework can be formalized with the following components: a set of states *𝒳*, a set of actions *𝒜*, a reward function *ℛ*, and a transition probability *𝒫*. The basic RL mechanism is as follows: at an arbitrary time *t*, the agent observes a state **x**_*t*_ ∈ *𝒳*, from the environment and outputs an action *a*_*t*_ ∈ *𝒜*. This action changes the environment and a new state **x**_*t*+1_ is observed. Upon transitioning to this new state, a reward *r*_*t+1*_ is presented from the environment to the agent. The process repeats either indefinitely or until a terminal state is reached. In RL, it is possible that the agent receives delayed reward information from the environment by unspecified time amounts.

### Policy and Value Functions

Two important concepts associated with the agent are the policy and value functions. The policy π is a function that maps a state **x**_*t*_ to an action *a_t_*, π : *𝒳* → *𝒜*. That is, the action taken by the agent is selected based on the agent’s policy. Moreover, the value function is a measure of the long-term performance of an agent following a policy π starting from a state **x**_*t*_. There are two types of value functions: a state-value function and an action-value function. The state-value function is defined as an expected value of a cumulative reward *R_t_*, which an agent receives when it starts in a particular state at time *t*, **x**_*t*_ and follows a policy π:


(1) $$V^{\pi }\left({\text{x}}_{t}\right)={𝔼}_{\pi }\left[R_{t}|{\text{x}}_{t}\right].$$

This state-value function indicates the expected cumulative reward that an agent can collect from a state **x**_*t*_. In addition, an action-value function considers the expected cumulative reward obtained by performing an action *a*_*t*_ while the agent is in the state **x**_*t*_ and following the policy π thereafter:


(2) $$Q^{\pi }\left({\text{x}}_{t},a_{t}\right)={𝔼}_{\pi }\left[R_{t}|{\text{x}}_{t},a_{t}\right].$$

A discounted infinite-horizon model is popularly chosen for the cumulative reward *R_t_*:


(3) $$R_{t}=\sum_{k=0}^{\infty }\gamma^{k}r_{t+k+1},0<\gamma <1,$$

where the discount factor γ provides emphasis on recently acquired reward values and prevents the function from growing unbounded as *k* → ∞.

The objective of RL is to find a good policy that maximizes the expected reward of all future actions given the current knowledge. By maximizing the rewards made available to an agent, the goal behavior can be realized. This duality is of course present by design and is commonly referred to as the *Reward Hypothesis*. Since the value function represents the expected cumulative reward given a policy, the optimal policy π*, can be obtained based on the value functions; a policy π is better than another policy π′ when the policy π gives a greater expected return than the policy π′. In other words, π ≥ π′ when *V*^π^ (**x**_*t*_) ≥ *V*^π′^ (**x**_*t*_) or *Q*^π^ (**x**_*t*_, *a*_*t*_) ≥ *Q*^π′^ (**x**_*t*_, *a*_*t*_) for all **x**_*t*_ ∈ *𝒳* and *a*_*t*_ ∈ *𝒜*. Therefore, the optimal state-value function *V*^π*^ (**x**_*t*_) is defined by,


(4) $$V^{\pi^{*}}\left({\text{x}}_{t}\right)=\max_{\pi }V^{\pi }\left({\text{x}}_{t}\right),$$

and the optimal action-value function *Q*^π*^ (**x**_*t*_, *a*_*t*_) can be obtained by,


(5) $$Q^{\pi^{*}}\left({\text{x}}_{t},a_{t}\right)=\max_{\pi }Q^{\pi }\left({\text{x}}_{t},a_{t}\right).$$

The following Bellman optimality equations are obtained by evaluating the Bellman equation for the optimal value function,


(6) $$V^{\pi^{*}}\left({\text{x}}_{t}\right)=\max_{a_{t}\in 𝒜\left(x_{t}\right)}\sum_{x_{t+1}}{𝒫}_{{\mathrm{xx}}^{\prime }}^{a}\left[{ℛ}_{{\mathrm{xx}}^{\prime }}^{a}+\gamma V^{\pi^{*}}\left({\text{x}}_{t+1}\right)\right],$$

(7) $$Q^{\pi^{*}}\left({\text{x}}_{t},a_{t}\right)=\sum_{x_{t+1}}{𝒫}_{{\mathrm{xx}}^{\prime }}^{a}\left[{ℛ}_{{\mathrm{xx}}^{\prime }}^{a}+\gamma \max_{a_{t+1}}Q^{\pi^{*}}\left({\text{x}}_{t+1},a_{t+1}\right)\right],$$

where ${𝒫}_{{\mathrm{xx}}^{\prime }}^{a}=P\left({\text{x}}_{t+1}={\text{x}}^{\prime }|{\text{x}}_{t}=\text{x},a_{t}=a\right)$ and ${ℛ}_{{\mathrm{xx}}^{\prime }}^{a}=𝔼\left[r_{t+1}|{\text{x}}_{t}=\text{x},a_{t}=a,{\text{x}}_{t+1}={\text{x}}^{\prime }\right]$. The solution to these Bellman optimality equations can be obtained using dynamic programming (DP) methods. However, this procedure is infeasible when the number of variables increases due to the exponential growth of the state space, the curse of dimensionality. In addition, solving this equation requires explicit knowledge of the environment including the state transition probability ${𝒫}_{{\mathrm{xx}}^{\prime }}^{a}$ and reward distribution ${ℛ}_{{\mathrm{xx}}^{\prime }}^{a}$ (Sutton and Barto, 1998).

### Functional Approximation of the Value Function and Policy

It is noteworthy that all published works on neural decoding within RLBMI use some form of functional approximation for either the value function or the policy. Therefore, in this section, we provide further details on how the functional approximation can be considered in RL. Moreover, this is another reason why we present in separate columns in Table 1, the RL base model and the function approximation strategies, along with the learning algorithms.

Various methods can approximately solve the Bellman optimality equations for each of the value functions. The approximate solutions often require far less time to resolve, with the added advantage of requiring less memory. The estimated value functions will allow comparisons between policies and thus guide the optimal policy search:


(8) $${\tilde{V}}^{\pi }\left({\text{x}}_{t}\right)=f_{v}\left({\text{x}}_{t};\theta_{f_{v}}\right),$$

(9) $${\tilde{Q}}^{\pi }\left({\text{x}}_{t},a_{t}\right)=f_{q}\left({\text{x}}_{t},a_{t};\theta_{f_{q}}\right),$$

where *f_v_* and *f_q_* represent arbitrary functions, and θ_*f*_*v*__ and θ_*f*_*q*__ are their corresponding parameters that define the function. Furthermore, following the same functional approximation strategy, the approximated policy can also be represented as follows:


(10) $$\pi :a_{t}\approx f_{\pi }\left({\text{x}}_{t};\theta_{f_{\pi }}\right),$$

where *f*_π_ and θ_*f*_π__ are an arbitrary function and its corresponding parameters, respectively. Therefore, to avoid high computational complexity and the need for having explicit knowledge of the environment including ${𝒫}_{{\mathrm{xx}}^{\prime }}^{a}$ and ${ℛ}_{{\mathrm{xx}}^{\prime }}^{a}$, this functional approximation strategy has been mainly considered in RLBMIs to model neural decoders.

While there exist various functional approximation methods, there are mainly two functional approximation methods that have been considered in RLBMI to approximate the value functions or policy. One is kernel basis expansion, and the other is artificial neural networks, specifically, feedforward networks and convolutional neural networks (CNNs).

#### Kernel Expansions

The basic idea of kernel methods is to nonlinearly map the input data to a high-dimensional feature space of vectors. Let *𝒳* be a nonempty set. For a positive definite function, κ : *𝒳* × *𝒳* → ℝ (Scholkopf and Smola, 2001; Liu et al., 2010), there exists a Hilbert space *ℋ* and a mapping ϕ : *𝒳* → *ℋ*, such that κ(**x**_1_, **x**_2_) = ⟨ϕ(**x**_1_), ϕ(**x**_2_)⟩. The inner product in the high-dimensional feature space can be calculated by evaluating the kernel function in the input space. Here, *ℋ* is called a reproducing kernel Hilbert space (RKHS) because it satisfies the following property,


(11) f(x)=⟨f,ϕ(x)⟩=⟨f,κ(x,⋅)⟩,∀f∈ℋ.

This property enables the transformation of conventional linear algorithms in the feature space into nonlinear systems without explicitly computing the inner product in the high-dimensional space. The function *f* can take the role of *f_v_*, *f_q_*, or *f*_π_ in RL as follows:


(12) $$f\left(x\right)=\sum_{i=1}^{n}\alpha_{i}\kappa \left({\text{x}}_{i},x\right),$$

where *n* corresponds to the number of available units to compute and α_*i*_ is the weighting factor for the unit centered at **x**_*i*_. In many cases, the number of available units corresponds to the number of data points that have been seen during training. We can think about kernel expansions as function approximators where the number of parameters can grow as more data become available.

#### Feedforward Neural Networks and Convolutional Neural Networks

An artificial feedforward neural network is composed of input, hidden (possibly multiple), and output layers, and each layer contains a certain number of units which are design parameters that depend on the problem set up. Let **x**^(ℓ)^ denote the activation vector at layer ℓ so that for a network with *L* layers, the input to the network is denoted as **x**^(0)^ and the output of the network as **x**^(*L*)^. The output of each unit in layer ℓ can be computed as follows:


(13) $$x_{j}^{\left(\ell \right)}=g_{j}^{\left(\ell \right)}\left(\sum_{i=1}^{d_{\ell -1}}w_{ij}^{\left(\ell \right)}x_{i}^{\left(\ell -1\right)}+b_{j}\right),$$

where $g_{j}^{\left(\ell \right)}$ represents an activation function, $w_{ij}^{\left(\ell \right)}$ are the weights connecting each layer’s units, *b_j_* is the bias term to be added, and *d*_ℓ_ resents the number of units in layer ℓ. The indexes *i* and *j* represent input to output units, respectively. In addition, $x_{i}^{\left(\ell -1\right)}$shows the *i*th input to the unit *j* and $x_{j}^{\left(\ell \right)}$ the unit’s output. Note that when *L = 1* and *g* is the identity function, this neural network corresponds to a linear function approximator.

A convolutional neural network is one type of artificial neural network where additional structure in the units can be used to group and restrict the weighted sum above to a convolution. For instance, an electroencephalogram (EEG) signal over a short time window has channel and time structure and can be seen as a single input array, similarly, an image can be seen as an input array with spatial structure and possibly also channel structure, RGB image as an example.

Along with these different function approximation strategies, various learning methods have been implemented in RLBMI. They are summarized in Table 1 and details are provided in the following sections, specifically section “Reinforcement Learning in Brain Machine Interfaces: Neural Decoding Algorithms.”

## Reinforcement Learning Brain Machine Interfaces: Basic Mechanism

**FIGURE 2:**
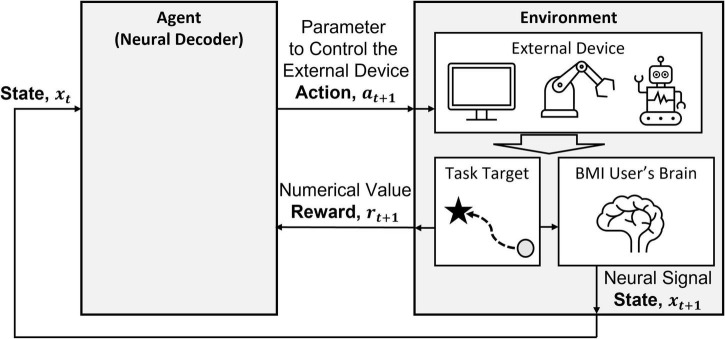
RLBMI architecture with labeled RL components. This figure is modified based on Figure 1 in DiGiovanna et al. (2009).

What makes RL most viable for BMIs is the ability of the agent to respond with continuous adaptations to a dynamic environment. In RLBMIs, the environment includes the subject, external device, and task-related information (Figure 2). RLBMIs consider the state of the environment **x**_*t*_ as the neural signals of the subject. The action *a_t_* generated from the agent is treated as a representation to control an external device, such as direction, position, or velocity. Moreover, the agent finds a mapping from the subject’s neural signal to the action, so the agent takes the role of the neural decoder.

In the RLBMI architecture, there are two intelligent systems: the BMI decoder in the agent and the user in the environment (DiGiovanna et al., 2009). The two intelligent systems learn co-adaptively based on closed-loop feedback. The agent updates the state of the environment, namely, the location of a cursor on a screen or a robotic arm’s position, based on the user’s neural activity and the received rewards. At the same time, the subject produces the corresponding brain activity. Through iterations, both systems learn how to earn rewards based on their joint behavior. The BMI decoder learns a control strategy based on the user’s neural state and performs actions in goal-directed tasks that update the action of the external device in the environment. In addition, the user learns the task based on the state of the external device. Notice that both systems act symbiotically by sharing the external device to complete their tasks, and this co-adaptation allows for continuous synergistic adaptation between the BMI decoder and the user even in changing environments.

### Environment in Reinforcement Learning Brain Machine Interface

Various experimental setups, including different types of subjects, external devices, and tasks, have been investigated to define the environment in RLBMIs, and Table 1 summarizes how each study is unique.

The reviewed studies showed variations of the subjects such as Sprague-Dawley rat, Bonnet Macaque, Rhesus Macaque, Marmoset monkey, and human. The neural signal type that has been used in RLBMI research also varies. However, our literature survey method identified that only two types of data acquisition technologies have been used with RLBMIs, namely, intracortical neural signals and EEG. Although these two types of signals differ in many ways, good performance of RLBMIs has been achieved with both neural signal modalities. In addition, it was also found that in some cases, the neural data were artificially generated. The simulated neuron’s activities may fail to capture all variations present in real-world scenarios but yield a viable method to showcase various theoretical properties or characteristics of an algorithm. Moreover, various dimensions of neural signals have been considered. The values listed inside of the parenthesis in “Neural signal types” in Table 1 contain details of the signal dimensions.

Different types of external devices have been employed in RLBMI experiments. A cursor on a 2D screen, a robotic arm, and a lever are the three different types of devices being reported. Moreover, numerous tasks have been investigated. A multi-target center-out reaching task and its variations, such as a multi-target reaching task and multi-target reaching and grasping task, have been the most commonly considered in RLBMIs, but go no-go task, lever pressing task, and obstacle avoidance task have also been applied.

### Agent in Reinforcement Learning Brain Machine Interface

Agent in RLBMI can be considered as a neural decoder since it provides a mapping from a state to action. Various RL algorithms have been considered in RLBMIs. We categorize the neural decoding algorithms based on the fundamental RL approaches each study considered. Q-learning, Watkin’s Q(λ), Attention-Gated Reinforcement Learning, and Actor-Critic are the main four RL algorithms considered in RLBMIs. The following section explains in further detail how each neural decoder works differently and points out each algorithm’s uniqueness.

In addition, each neural decoder’s reported performance is also summarized. We categorize its performance based on task type and open- or closed-loop experimental setups. It is notable that even though most of the studies implement RLBMI in open-loop setups, similar types of neural decoders have been implemented in both open- and closed-loop experiments. The open-loop experiments allow more resource intensive investigations, yet the closed-loop experiments provide the most applicable setup for real-world deployments.

## Reinforcement Learning in Brain Machine Interfaces: Neural Decoding Algorithms

Table 1 provides an itemized summary of reviewed neural decoders integrated in RLBMI. This section provides further details of each neural decoder, along with Table 1. We first categorize each neural decoder based on the RL base model in sections “Approximation of the Action-Value Function, Q” and “Actor-Critic.” We then list learning algorithms for each model under their corresponding subsections. Specific neural signal type is identified and the type of task, which the external device needed to complete, is summarized. In addition, key-reported performances are listed in terms of success rates.

For the best comparison of overall reviewed neural decoders in RLBMI, we chose success rates as the evaluation metric. Since the function approximation algorithms are typically applied to approximate the value functions in RLBMI, it is common to show how the value function is estimated to evaluate the neural decoder’s performance. However, the estimated value is not always directly associated with how an actual movement is selected. Furthermore, confusion matrix and precision-recall curves are commonly considered evaluation metrics in typical classification tasks, but not all reviewed studies report them. Note that these metrics are only suitable when a single step reaching task is considered because an action, a choice of direction that can match a class label, happens at each step in multi-step tasks. In addition, we only report the best performances in each study. Generalization of the reported performance is still limited due to neural and measurement variability. Each study reports the neural decoder’s performance on each subject and session separately. Since each study has a different number of subjects and recording sessions, we describe the best reported performance.

### Approximation of the Action-Value Function, Q

A recently published study introduces how a linear approximation of the action-value function *Q* can be used to detect Chinese symbols under the P300 brain–computer interface paradigm (Huang et al., 2022). The P300 brain–computer interface paradigm uses a unique setting that requires stimulations to produce synchronization of EEG patterns. This study uses different visual stimulations to represent each row and column that can be associated with a symbol location in a 6 × 6 (row × column) display. A linear relationship is used to approximate the action-value function, *Q* = θ^*T*^ **x**, where θ is a coefficient vector, and **x** is constructed from a *d*-dimensional feature vector based on the EEG epoch. The θ values are optimized by minimizing the difference between the expected and the actual *Q* values, $Q^{*}-\tilde{Q}$. For an action selection strategy, an upper confidence bound (UCB) is used. This study also provides transferred P300 linear upper confidence bound (TPLUCB), by transferring θ information from different subjects to a new subject. PLUCB and TPLUCB showed improved performance over a conventional algorithm called stepwise linear discriminant analysis (SWLDA); their reported overall symbol accuracies are 80.4 ± 12.8% and 79.6 ± 14%, respectively.

### Q-learning and Its Variations

Temporal difference (TD) learning is an incremental learning method specialized for multi-step prediction problems. It provides an efficient learning procedure that can be applied to RL. TD learning allows learning directly from new experiences without having a model of the environment. In addition, it employs temporal difference error, in composition with previous estimations, to provide updates to the current predictor (Sutton, 1988).

Q-learning is an off-policy TD algorithm based on the following incremental TD update rule for the action-value function.


(14) $$Q\left({\text{x}}_{t},a_{t}\right)\leftarrow Q\left({\text{x}}_{t},a_{t}\right)+\eta [r_{t+1}+\gamma \max_{a}Q\left({\text{x}}_{t+1},a\right)-Q\left({\text{x}}_{t},a_{t}\right)],$$

where η and γ are the step-size and discount factors, respectively, and η, γ ∈[0,1]. The current action *a_t_* is selected based on a policy derived from the current *Q*(**x**_*t*_, *a*_*t*_), and ϵ-greedy is a commonly considered policy. Despite the policy, this update rule allows selecting the next action *a*_*t+1*_, which results in the greatest valuation of *Q* given the state and action pair. Q-learning does not require a model of the environment to converge upon an optimal policy and is, therefore, invaluable in stochastic and dynamical learning situations.

The Q(λ) algorithm is an extension of Q-learning by adding the eligibility trace λ, which allows learning, based on a sequence of actions selected. Although there are two different Q(λ) algorithms, including Watkins’ Q(λ) (Watkins, 1989) and Peng’s Q(λ) (Peng and Williams, 1996), the RLBMI studies showed a specific focus on Watkin’s Q(λ) algorithm. Watkin’s Q(λ) algorithm uses the following cost function *J_t_*:


(15) $$J_{t}=\frac{1}{2}{\left(TDerror_{t}^{\lambda }\right)}^{2},$$

(16) $$TDerror_{t}^{\lambda }=TDerror_{t}+\sum_{n=1}^{T-1}{\left(\gamma \lambda \right)}^{n}TDerror_{t+n},$$

(17) $$TDerror_{t}=r_{t+1}+\gamma Q\left({\text{x}}_{t+1},a_{t+1}\right)-Q\left({\text{x}}_{t},a_{t}\right),$$

where *T* is the length of a trial. Its update rule is derived by $\frac{\partial J_{t}}{\partial Q\left(x_{t},a_{t}\right)}=0$.

Attention-Gated Reinforcement Learning was introduced as a biologically realistic learning scheme by integrating feedback connections, called attention effects, and synaptic plasticity (Roelfsema and van Ooyen, 2005). Attention-Gated Reinforcement Learning is a policy-based learning method with an instantaneous reward. Two unique components of Attention-Gated Reinforcement Learning are global error signal δ, which reflects changes in reward expectancy, and an attention signal, which feeds back from the output layer to the previous layers. The global error signal δ is defined in such a way, that it increases learning when unexpected actions are taken. Another key difference between the Attention-Gated Reinforcement Learning is a form of policy π for which the units in the output layer engage in a competition. That is, the new form of policy introduces that in each iteration, one output unit is selected, based on the stochastic Softmax rule, and only the winning unit is updated (Roelfsema and van Ooyen, 2005).

It is notable that compared to Q(λ) algorithms, Attention-Gated Reinforcement Learning considers the same mechanisms of state and action relations; that is, a neural signal is treated as an input state, **x**_*t*_, and the output is represented as the action, *a_t_*, to control an external device. Moreover, the Attention-Gated Reinforcement Learning network is set to estimate the action-value function, *Q*. The unique difference in the Attention-Gated Reinforcement Learning network is that a new form of policy is applied to select one corresponding action.

#### Q-Learning via Kernel Temporal Difference(λ)

The value functions can be estimated adaptively using the TD(λ) algorithm, which approximates the value functions using a linear function approximator. However, this may be a limitation in practice. A nonlinear variant of the TD algorithm, called Kernel Temporal Difference(λ), was introduced by integrating kernel methods (Bae et al., 2011, 2015).

Bae et al. (2011) showed how the action-value function *Q* can be approximated using Kernel Temporal Difference(λ) in Q-learning, ${\tilde{Q}}^{\pi }\left({\text{x}}_{t},a_{t}\right)=f\left({\text{x}}_{t},a_{t};\theta_{f}\right)$. The function *f* can be optimized using the following update rule:


(18) $$f\leftarrow f+\eta \sum_{i=1}^{m}\Delta {\tilde{f}}_{i},$$

(19) $$\Delta {\tilde{f}}_{i}=\left(r_{i+1}+\left\langle f,\gamma \phi \left({\text{x}}_{i+1}\right)-\phi \left({\text{x}}_{i}\right)\right\rangle \right)\sum_{k=1}^{i}\lambda^{i-k}\phi \left({\text{x}}_{k}\right).$$

**FIGURE 3:**
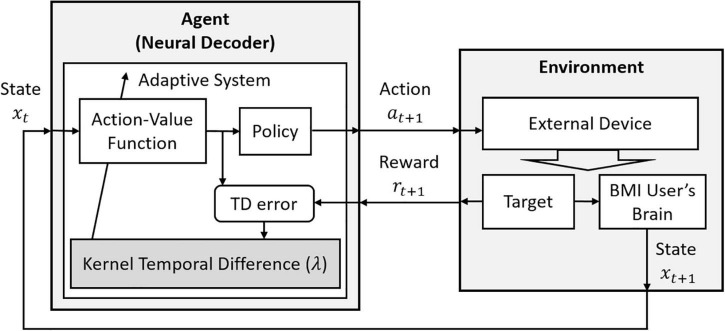
The decoding structure of RLBMI using a *Q*-learning via Kernel Temporal Difference(λ). This figure is modified based on Figure 1 in Bae et al. (2015).

Here, η is the stepsize, and *m* is the length of a trial. We should note that differently from Q(λ), this algorithm uses the eligibility trace λ as in TD(λ) (Sutton, 1988). That is, the λ value is not set to zero depending on the chosen greedy policy but takes a main role as a memory to trace more recent trials. Figure 3 shows how this algorithm can be considered in the basic RL structure.

Bae et al. (2011) showed that using female Bonnet Macaque’s intracortical recordings, this algorithm properly finds matching directions on a 2-target center-out reaching task after 2 epochs of training. The application of Kernel Temporal Difference(λ) was extended and a convergence property was explained in Bae et al. (2015). This study investigated the algorithm’s performance on various setups in open-loop experiments and presented results from closed-loop RLBMI experiments, using monkey’s intracortical signals. Considering that most of the reviewed studies implemented RL-based neural decoding algorithms on a single-step task, which allows one step from the initial location to the target, a distinctive feature of this study is that it investigated multi-step reaching tasks, as well. In addition, the best performance on the closed-loop 2-target reaching task to control a robotic arm showed 90% accuracy.

#### Q-Learning via Correntropy Kernel Temporal Difference

A new cost function, called Correntropy, has been integrated in Kernel Temporal Difference, to address possible issues under noise-corrupted environments (Bae et al., 2014). Highly noise-corrupted environments lead to difficulties in learning, and this may result in failure to obtain the desired behavior of the agent. The generalized correlation function, Correntropy, was first introduced by Liu et al. (2007). Correntropy is defined in terms of inner products of vectors in the kernel feature space,


(20) $$Correntropy\left({\text{X}}_{1},{\text{X}}_{2}\right)=𝔼\left[\kappa \left({\text{X}}_{1}-{\text{X}}_{2}\right)\right],$$

where ***X***_*1*_ and **X**_2_ represent two random variables, and κ is a translation invariant kernel. When Correntropy is set as a cost function in Kernel Temporal Difference(λ), Q-learning via Correntropy Kernel Temporal Difference approximates the action-value function *Q* for an action *k* in the following way;


(21) $$\tilde{Q}\left({\text{x}}_{t},a_{t}=k\right)=\eta \sum_{i=1}^{t-1}e^{-\left(\frac{TDerror_{i}^{2}}{2h_{c}^{2}}\right)}TDerror_{i}I_{i}^{k}\kappa \left({\text{x}}_{t},{\text{x}}_{i}\right),$$

where η is the stepsize, *h_c_* is the Correntropy kernel size, and *TDerror_i_* denotes a Temporal Difference error defined as *TDerror*_*i*_ = *r*_*i*+1_ + γmax_*a*_ *Q* (**x**_*i*+1_, *a*) − *Q*(**x**_*i*_, *a*_*i*_ = *k*). Recall that the reward *r*_*i + 1*_ corresponds to the action selected by the current policy with input **x**_*i*_ because it is assumed that this action causes the next input state **x**_*i*+1_. Here, $I_{i}^{k}$ is an indicator vector with the same size as the number of outputs; only the kth entry of the vector is set to 1, and the rest of the entries are 0. The selection of the action unit *k* at time *i* can be based on an ϵ-greedy method. Therefore, only the parameter vector corresponding to the winning action gets updated. Correntropy Kernel Temporal Differences showed slightly faster learning speed than Kernel Temporal Difference(λ = 0) when intracortical recordings from a female Bonnet Macaque were decoded to control a cursor on a screen in a 4-target center-out reaching task. In addition, interestingly, more balanced learning through four different targets was observed, compared to Kernel Temporal Difference(λ = 0), and this may bring a potential benefit to the closed-loop RLBMIs.

#### Dueling Deep Q Networks

Although there have been dramatic expansions in deep RL studies, which combine RL and deep learning, the application of deep RL in BMIs still lacks. Zhang et al. (2019b) is a unique study introducing the application of Dueling Deep Q Networks (Wang Z. et al., 2015) to classify different neural patterns associated with six different behaviors. In addition, considering most of RLBMIs have used intracortical neural signals, this study is distinctive from other studies, by using EEG. It should be noted that due to the challenges of EEG, lower signal-to-noise ratio and spatial resolution than the intracortical recordings, the imagery actions related to typing commands are not directly associated. For instance, to make a robot move forward, the subject should imagine upward, and for turning left, the subject should imagine downward, etc.

Dueling Deep Q Networks use a special deep network architecture composed of a set of convolutional layers followed by two streams of fully connected layers. The basic idea of Dueling Deep Q Networks is to estimate the action-value function *Q* in composition with the state-value function *V* and the advantage function, *A*^π^(**x**_*t*_, *a*_*t*_) = *Q*^π^ (**x**_*t*_, *a*_*t*_) − *V*^π^(**x**_*t*_), using the deep network architecture:


(22) $$Q\left({\text{x}}_{t},a_{t};\theta ,w,v\right)=V\left({\text{x}}_{t};\theta ,v\right)+\left(A\left({\text{x}}_{t},a_{t};\theta ,w\right)-\max_{a\in \left|𝒜\right|}A\left({\text{x}}_{t},a;\theta ,w\right)\right),$$

where θ represents the parameters of the convolutional layers, *w* and *v* are the parameters of the two streams of the fully connected layers, respectively. That is, after the input neural representation passes through the convolutional layers, the output of the convolutional layer splits into two different fully connected networks, which represent the state-value function *V* and the advantage function *A*, separately. Once the state-value function *V* and the advantage function *A* are obtained, the action-value function can be computed. Based on seven healthy human EEGs, this algorithm reached average classification accuracy of 93.63%.

#### Watkin’s Q(λ) With Conventional Artificial Neural Networks

Watkin’s Q(λ) has been considered to find the optimal policy π*. Depending on BMI applications, various strategies have been applied to approximate the action-value function *Q* in Watkin’s Q(λ). For example, an Artificial Neural Network was implemented in DiGiovanna et al. (2007b) and Sanchez et al. (2011), and a Time Delayed Neural Network (TDNN) was applied in DiGiovanna et al. (2007a, 2009) and Tarigoppula et al. (2012).

DiGiovanna et al. (2007b) investigated single-layer perceptron, multilayer perceptron with linear outputs, and multilayer perceptron with nonlinear outputs in go no-go task to control a robotic arm’s movement using a rat’s intracortical signals. Interestingly, this study combines a supervised learning algorithm, called the Multiple Paired Forward Inverse Model, to decide whether the robotic arm’s moving direction is either to the left or right. In the case of the single-layer perceptron, in the closed-loop experiment, the neural decoder reached 93.7% performance accuracy in the first session containing 16 trials, where 8 trials are for the left target and the rest for the right target.

Sanchez et al. (2011) used multilayer perceptron with back propagation to estimate the action-value function with Watkin’s Q(λ). In an open-loop experiment, this study shows that the neural decoder can properly find a bonnet macaque’s intracortical signal to action directions in an 8-target center-out reaching task on a 2D screen.

In addition, DiGiovanna et al. (2007a, 2009) are from the same main authors, and these studies follow the same experimental paradigm and decoding algorithm; both showed that using a TDNN with backpropagation in Watkin’s Q(λ) to estimate the action-value function *Q*, a rat’s intracortical signal can be successfully decoded to control a robotic arm.

The work of Tarigoppula et al. (2012) investigated the properties of neurons that help to obtain a reasonable performance of the neural decoder. The authors tested a computational spiking neuron model, named Izhikevich neuron model (Izhikevich, 2004), on an 8-target center-out reaching task to validate the correlation with Izhikevich-tuning depth and the Q(λ) learning’s success rate. The authors defined the Izhikevich-tuning depth as *a*/*b*, where the current inputs to the Izhikevich neuron are noted as *I* = *a* × (*weightage of a neuron*) + *b*, and *a* and b are variables to be chosen. The defined tuning depth explains that the behavior of the neuron model is influenced by the ratio of modulated input current, indicated in *a*, and the baseline input current, represented as *b*. In addition, a TDNN with backpropagation was used in Watkin’s Q(λ) to approximate the action-value function. Different depth values are investigated with 80 neurons, and it is shown that when Izhikevich-tuning depth is over 0.75, the RL agent provides a success rate of over 95%.

#### Attention-Gated Reinforcement Learning

Attention-Gated Reinforcement Learning has been applied in RLBMI (Wang Y. et al., 2015 and Shen et al., 2020). When a three-layer neural network is considered, the weights between the input and hidden units $w_{ij}^{\left(1\right)}$ and hidden and output units $w_{jk}^{\left(2\right)}$ are updated based on the error backpropagation rule (Bishop, 1995) as follows:


(23) $$w_{ij}^{\left(1\right)}\leftarrow w_{ij}^{\left(1\right)}+\eta_{1}x_{i}^{\left(0\right)}x_{j}^{\left(1\right)}f\left(\delta \right)\left(1-x_{j}^{\left(1\right)}\right)\sum_{k=1}^{C}x_{k}^{\left(2\right)}w_{jk}^{\left(2\right)},$$

(24) $$w_{jk}^{\left(2\right)}\leftarrow w_{jk}^{\left(2\right)}+\eta_{2}x_{j}^{\left(1\right)}x_{k}^{\left(2\right)}f\left(\delta \right),$$

where η_1_ and η_2_ are the stepsizes, $x_{i}^{\left(0\right)},x_{j}^{\left(1\right)},$ and $x_{k}^{\left(2\right)}$ represent the input, hidden, and output units, respectively. *C* shows the number of the hidden units. Here, the input unit, $x_{i}^{\left(0\right)}$, is the representation of the neural signals, and the output unit $x_{k}^{\left(2\right)}$ is the action-value function representing one class of action. In addition, the expansive function *f*(δ) is a function of the global error. It will be described in the following paragraph, how Wang Y. et al. (2015) study sets this expansive function as an example.

One requirement of Attention-Gated Reinforcement Learning is the instantaneous reward. Thus, the approximated instantaneous reward is commonly considered. For example, in Wang Y. et al. (2015), the instantaneous reward was approximated based on the distance differences of the moving cursor as follows:


(25) rt+1={11+e-αΔdt, Δdt≠0       1,       Δdt=0,π(st)=ahorar,

where α is a scaling factor, which was set to 20, and Δ*d*_*t*_ is the distance between the position of the moving cursor at time *t* and *t + 1*. *a_h_* and *a_r_* correspond to the monkey’s actions, which are holding the joystick and resting, respectively. Once the agent receives the instantaneous reward, the global error signal can be defined. Wang Y. et al. (2015) sets the global error signal as follows:


(26) $$\delta_{t}=\left[2-\text{Pr}\left(x_{k,t}^{\left(2\right)}=1\right)\right]r_{t+1}-1.$$

Here, $\text{Pr}\;\left(x_{k,t}^{\left(2\right)}=1\right)$is the probability that the output unit *k* at time *t* is the winning unit. The definition of the global error signal leads to defining the expansive function. In Wang Y. et al. (2015), the expansive function is set as follows:


(27) f(δt)={δt1-δt+β, δt≥0         δt,           δt<0,

where β is the scaling factor, which was set as 10^−4^. This expansive function is used in the updating rule.

In Wang Y. et al. (2015), a male Rhesus Macaque’s intracortical signals from the primary motor cortex (M1) were recorded while it was moving a joystick. The joystick’s corresponding movement was displayed on a 2D screen as a cursor location. Its intracortical signals are input to the Attention-Gated Reinforcement Learning model to reach four different target locations. This experiment allowed seven different actions including up, down, left, and right position holding of *y*-axis, position holding of *x*-axis, and resting. After applying 4 days of data (days 1, 2, 3, and 6), allowing 40 min recordings per day, their neural decoder reached an average target acquisition rate of 90.16%.

In addition, by modeling reward, based on the medial prefrontal cortex (mPFC) from rates using a support vector machine, Shen et al. (2020) showed the possibility of using Attention-Gated Reinforcement Learning in autonomous RLBMI that can self-evaluate the external device’s behavior. After the introduction of successful implementation of Attention-Gated Reinforcement Learning, variants of Attention-Gated Reinforcement Learning have been introduced in RLBMIs, which are described in the following subsections.

#### Transfer Learning Mini Batch Attention-Gated Reinforcement Learning

The main departure of Zhang et al. (2020) from Wang Y. et al. (2015) is the incorporation of transfer learning and mini-batch training to alleviate degradation of performance on the agent due to neural plasticity; i.e., changing neural patterns over time associated with the same action. A principal component analysis (PCA)-based domain adaptation was used as the form of transfer learning, which projects the previously observed neural data and current data to a shared feature space that reduces the differences between them. After that, at each iteration, a mini-batch of samples, the last *N* samples, is used to update the Attention-Gated Reinforcement Learning weights.

In Zhang et al. (2020), the error signal δ_*t*_ was defined in the following way,


(28) $$\delta_{t}=r_{t}-𝔼\left[r_{t}\right],$$

where 𝔼[*r*_*t*_] represents the expected reward based on the agent’s policy. The expansive function is defined as the same in Wang Y. et al. (2015) by setting β = 0. Note that Shen et al. (2020) and Zhang et al. (2020) both alter the method proposed in Wang Y. et al. (2015) with a slightly different error signal and expansive function, while Shen et al. (2020) also incorporates a reward model and Zhang et al. (2020) introduces transfer learning and mini-batch concepts.

Zhang et al. (2020) used intracortical recordings from two adult male Rhesus Macaques while they are performing 3-target reaching and grasping tasks. The neural decoder showed a success rate of approximately 90% for both monkeys.

#### Maximum Correntropy-Based Attention-Gated Reinforcement Learning

The same group as Wang Y. et al. (2015) integrated Correntropy (Liu et al., 2007) as a cost function to obtain robust Attention-Gated Reinforcement Learning performance (Liu et al., 2007). By taking the Correntropy as a cost function in Attention-Gated Reinforcement Learning, the updating rule introduced in Attention-Gated Reinforcement Learning is modified as follows:


(29) $$w_{ij}^{\left(1\right)}\leftarrow w_{ij}^{\left(1\right)}+\eta_{1}x_{i}^{\left(0\right)}x_{j}^{\left(1\right)}\kappa \left(\delta \right)f\left(\delta \right)\left(1-x_{j}^{\left(1\right)}\right)w_{jk}^{\left(2\right)},$$

(30) $$w_{jk}^{\left(2\right)}\leftarrow w_{jk}^{\left(2\right)}+\eta_{2}x_{j}^{\left(1\right)}\kappa \left(\delta \right)f\left(\delta \right),$$

where κ is the Correntropy kernel function, and κ (δ) represents the kernel value on the error signal δ. This yields the following Correntropy definition:


(31) Correntropy=𝔼[κ(δ)].

When a Rhesus Macaque was performing a 4-target obstacle task on a 2D screen by moving a joystick, the monkey’s premotor cortex signals were recorded. The new cost function in Attention-Gated Reinforcement Learning allowed an improved success rate of decoding the monkey’s neural intention by more than 20% (from 44.63 to 68.79%) compared to Attention-Gated Reinforcement Learning (Li et al., 2016).

#### Quantized Attention-Gated Kernel Reinforcement Learning

Moreover, to address the issue of local minima entrapment on the multilayer perceptron, employed in Attention-Gated Reinforcement Learning, the same group, as Wang Y. et al. (2015), extended Attention-Gated Reinforcement Learning by integrating kernel methods (Wang et al., 2017). That is, the action-value function was approximated by a superposition of kernels as in kernel methods,


(32) $${\tilde{Q}}_{k}\left({\text{x}}_{t}\right)=\sum_{k=1}^{t-1}\eta f\left(\delta \right)\;\langle \phi \left({\text{x}}_{t}\right),\phi \left({\text{x}}_{k}\right)\rangle$$

$$=\sum_{k=1}^{t-1}\eta f\left(\delta \right)\kappa \left({\text{x}}_{t},{\text{x}}_{k}\right),$$

where η is the stepsize, *f*(δ) is the expansive function, and κ is the kernel function. In addition to the introduction of the kernel method, Wang et al. (2017) applied a quantization method (Chen et al., 2012) to avoid the linear growth of the computational complexity.

A male Rhesus Macaque’s intracortical signals from both M1 and primate dorsal premotor cortex (PMd) were decoded to a cursor location to perform a 4-target obstacle avoidance task. The monkey was moving a joystick to control the cursor location displayed on a 2D screen. Authors investigated various learning scenarios and average success rates of 80.83 ± 10.3% were reported. It is remarkable that although the value of the success rate seems lower than the other reported studies including Wang Y. et al. (2015) and Zhang et al. (2020), considering the complexity of the task type, obstacle avoidance tasks, this success rate is significant.

#### Clustering-Based Kernel Reinforcement Learning

Zhang et al. (2018, 2019a) extended Quantized Attention-Gated Kernel Reinforcement Learning by introducing the concept of data clustering. The data clustering considers only the selected subspace of RKHS to compute the action-value function. Moreover, the weight update is applied only to the nearest cluster to the chosen action.

Zhang et al. (2018) provide a proof of concept of the clustering-based kernel RL approach in RLBMIs by using simulated neurons. This study shows decoding performance improvement of this approach (99.8 ± 6.6%) compared to Quantized Attention-Gated Kernel Reinforcement Learning (97.8 ± 8.8%). Moreover, Zhang et al. (2019a) use a male Macaque’s intracortical recordings from M1 to control a robotic arm. It shows that with a relatively small number of kernels (approximately 800 kernels) compared to Quantized Attention-Gated Kernel Reinforcement Learning (approximately 3500 kernels), their proposed approach can reach similar decoding accuracy to Quantized Attention-Gated Kernel Reinforcement Learning. With sufficient RL training, higher accuracy (94.3 ± 0.9%) than Quantized Attention-Gated Kernel Reinforcement Learning (91.8 ± 3.4%) is observed. In addition (Zhang and Wang, 2019), shows the efficiency of using weight transfer to lead the agent’s quick adaptation to a similar task. This study validates how the acquired decoding knowledge from one task can be effectively transferred to a similar task, by first training the RL agent to one-level pressing tasks to two-level pressing tasks using three simulated neurons.

### Actor-Critic

**FIGURE 4:**
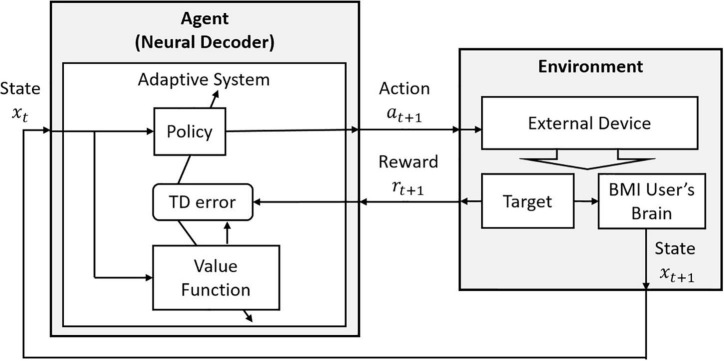
The decoding structure of RLBMI using the Actor-Critic. This figure is modified based on Figure 6.15 in Sutton and Barto (1998).

Actor-Critic contains two separate structures: one takes a role as a policy since it selects an action based on a given state, and this structure is called an *Actor*. Another structure estimates the value function, and it is known as a *Critic*. In addition, compared to Q-learning, Actor-Critic is an on-policy algorithm. This means that the Critic always follows a fixed policy provided by the Actor. In RLBMIs, the conventional Actor-Critic model has been extended to directly communicate with the user’s neural signals. That is, at each time, the Actor selects an action *a_t_* based on the user’s neural signal **x**_*t*_ and the Critic provides a Temporal Difference error to update the policy in the Actor, based on the estimated value function in the Critic (Figure 4).

#### Actor-Critic With Artificial Neural Networks

In Mahmoudi and Sanchez (2011), the policy in the Actor was modeled using a TDNN, and with the objective function, this results in the following update rule:


(33) $$\theta \leftarrow \theta +\eta TDerror_{t}\Psi \left({\text{x}}_{t}\right),$$

where η is the stepsize, the temporal difference error is defined as an instantaneous error, *TDerror*_*t*_ = γ*Q*_*t*_ − *Q*_*t* + 1_, and Ψ(**x**_*t*_) represents the projected M1 neural state in the feature space. In this study, the Actor tries to optimize the parameters of the policy π(**x**_*t*_, *a*_*t*_ | θ) to maximize the average expected rewards; that is, θ* = *argmax*_π_*J* (θ), where $J\left(\theta \right)=\frac{1}{T}\sum_{t=0}^{T-1}Q_{t}$, and *T* is the number of steps considered.

A uniqueness of the approach of Mahmoudi and Sanchez (2011) is that the reward value was assigned directly from the recorded activities from Nucleus Accumbens (NAcc). Moreover, the neural state was used from the intracortical neural signals at M1. It is notable that Mahmoudi and Sanchez (2011) follow the same experimental setup introduced in DiGiovanna et al. (2007a, 2009). Although the authors found that NAcc obtains a rich representation of goal information, it is still challenging to decide how to assign a specific reward value from the acquired neural population information at NAcc.

#### Actor-Critic With Hebbian Reinforcement Learning

Although integration of Hebbian reinforcement learning to train Actor was first introduced and implemented in BMIs in Pohlmeyer et al. (2012), Mahmoudi et al. (2013) provides details on how the Hebbian reinforcement learning can be used to train the Actor. Consider a probability mass function *g* written as,


(34) $$g\left(\rho ,w_{ij}^{\left(1\right)},x_{i}^{\left(0\right)}\right)=\text{Pr}\left(x_{j}^{\left(1\right)}=\rho |w_{ij}^{\left(1\right)},x_{i}^{\left(0\right)}\right),$$

where the node *j* takes a certain value ρ. The input $x_{i}^{\left(0\right)}$ from node *i* through synaptic weight $w_{ij}^{\left(1\right)}$ generates the output $x_{j}^{\left(1\right)}$. The weights can be updated based on Hebb’s rule (Trappenberg, 2004), which follows the activity-dependent features of synaptic plasticity. By extending the Hebbian reinforcement learning, the authors introduced the following update rule:


(35) wij(1)←wij(1)+ηr+(xj(1)-pj)xi(0)+η-(1-r)(1-xj(1)-pj)xi(0),

where η^+^ and η^−^ are separate step sizes corresponding to reward and penalty components, respectively, and *p_j_* is an output state of the node *j*. In addition to Pohlmeyer et al. (2012, 2014), Mahmoudi et al. (2013), and Prins et al. (2014) use the same learning algorithm for the neural decoder. Note that in these studies differently from Mahmoudi and Sanchez (2011), signals acquired from NAcc were not used to provide the reward values to the Critic.

Mahmoudi et al. (2013) showed that the neural decoder can adapt to episodic tasks over time. By simulating neuron activities based on the standard Izhikevich neuron model, the authors first start with 2-target reaching task and then expanded to 4-target reaching task, and it was shown that the neural decoder can adapt to the changing environment over time and reach 100% success rates in less than a total of 75 trials. Moreover, in an open-loop experiment, based on two Marmoset monkeys’ intracortical neural signals, recorded at M1, the neural decoder reached over 95% success rates after 20 trials for both monkeys.

In addition, Pohlmeyer et al. (2012, 2014) are from the same authors, and these two studies share the same experimental paradigm, yet Pohlmeyer et al. (2014) presents an expanded study from Pohlmeyer et al. (2012). Pohlmeyer et al. (2012) used a fully connected, three-layer, feedforward neural network integrated with the Hebbian reinforcement learning algorithm in the Actor. The Actor-Critic algorithm showed an average 90% success rate over eight sessions for the first 50 trials by decoding a Marmoset monkey’s intracortical signals from M1 and NAcc. Pohlmeyer et al. (2014) used the same subjects and followed the same experimental paradigm as Mahmoudi et al. (2013), but it was extended to closed-loop experiments. Interestingly, even for the closed-loop experiments, a similar performance was reported; over 90% success rates for both monkeys and four sessions.

Moreover, Prins et al. (2014) investigated how Critic’s feedback influences the overall performance of the Actor-Critic with the Hebbian reinforcement learning. When the Critic was able to provide 90% accurate feedback value using the Hebbian reinforcement learning approach to Actor, performance improvements on the policy were observed (from 77 to 83%).

Furthermore, Roset et al. (2014) provided an experimental test bed for using the Actor-Critic with the Hebbian reinforcement learning to control a functional electrical stimulation device on a subject with chronic spinal cord injury using EEG. For four closed-loop sessions, performances on both Actors and Critics reached around 65%. In this study, it is notable that the Critic used the detected error-related potential (ErrP) to input binary feedback to the Actor.

## Discussion

BMIs have great potential to help paralyzed individuals regain movement capabilities. RL provides its unique learning mechanism based on trial-and-error paired with rewards that enable active exploration of the environment. The indirect learning guidance, given in terms of a reward signal, which can be directly obtained from the user’s brain activity, allows RL techniques to be seamlessly integrated into a larger variety of tasks allowing for more versatile and realistic BMIs.

From the literature, we covered in this article, we can see that the neural decoders in RLBMIs can be categorized by the functional approximation approaches and learning strategies on their key RL components. Functional approximators provide estimated value function and policy in RL, by integrating with different learning methods. Therefore, when considering neural decoders in RLBMI, it is required first to decide on the RL base model that can determine how certain value functions and/or policies will be approximated. For example, if Q-learning is chosen, action-value function is approximated based on the state and reward values. Second, a type of function approximator should be chosen and then an appropriate learning method must be selected. Depending on the choice of the function approximation method, different parameters need to be tuned and factors, such as computational complexity, must be considered.

Although studies show that RL has great potential for BMI applications, RLBMI applications are still limited. Our literature review shows that two main RL base models have been considered in RLBMIs: Q-learning and Actor-Critic. Although we separately listed Q-learning, Watkin’s Q(λ), and Attention-Gated Reinforcement Learning in Table 1, Watkin’s Q(λ) and Attention-Gated Reinforcement Learning are Q-learning variants, as we mentioned in section “Q-learning and Its Variations.” We find that most of the studies used Q-learning. We consider one of the main reasons behind this is that Q-learning allows the simplest and reasonably effective learning format for neural decoders. Both Q-learning and Actor-Critic use a measure of the value function. However, Q-learning specifically considers the action-value function, but Actor-Critic typically uses the state-value function in the Critic. The main difference between Q-learning and Actor-Critic is that Actor-Critic has an additional component, called an Actor that updates the policy based on exploration. It is known that this policy improvement strategy from the Actor, by using the estimated value function from the Critic, provides more stable and faster learning than Q-learning (Sutton and Barto, 1998). However, from the reviewed RLBMI studies, no critical performance differences were observed. This may be due to other factors at play such as the functional approximation and learning models and the complexity of the experimental setup and neural signals. From the reported studies, it is limited to conclude which RL base model is more suitable for BMIs since no study has shown comparisons of the two different RL base models. Each study selects one RL base model and has its own experimental setup and subjects. Some studies show comparisons of the performance of different functional approximation and learning models, but they use the same RL base model.

Learning speed on RLBMIs depends on the complexity of the tasks and neural patterns from the subject. The choice of functional approximation method determines the computational complexity and number of parameters to optimize. This can influence the speed of learning and generalization capabilities. When selecting the functional approximation algorithm, the RLBMI designer should be aware of each method’s characteristics. An artificial neural network is the most well-known machine-learning model and perhaps the easiest one to implement, thanks to the availability of well-developed machine-learning toolboxes and libraries for mainstream coding languages. However, when artificial neural networks are considered, strategies to overcome local minima or saddle points and to initialize weights should be addressed, and the structure of the neural network, including the type and number of layers and units per layer, should be carefully selected along with other hyperparameters including the step size used for training with gradient descent.

Furthermore, kernel methods allow an effective way of computation, allowing nonlinear approximation in input space, but linear computation in the feature space, RKHS. Depending on the use, convergence can be also guaranteed. However, when kernel methods are considered, one must factor in how the implementation can handle a potentially increasing number of kernel units. This requires the incorporation of additional methods that control the growth of the number of kernel centers in the structure.

In addition, characteristics of the learning method should be also considered when designing neural decoders in RLBMIs. For instance, Correntropy provides robust performance in the presence of outliers, but it requires tuning of additional hyperparameters, such as the Correntropy kernel size, and a proper understanding of the environment is required, since Correntropy brings benefits to the performance under certain conditions, including cases where there are highly noisy neural signals, and the reward values are corrupted. Furthermore, batch approaches demand investigating what is the optimal batch size and a suitable update strategy. In RLBMI, further investigation of functional approximation and learning methods should be conducted. In the reported studies, the choice of the function approximation method is still limited, and the effects of certain learning strategies on the selected function approximation method have not been fully understood.

Although similar tasks have been considered in various studies, it is still limited to conclude which neural decoder provides the best performance due to the subject variations and differences in the experimental setup. Ideally, a neural decoder in RLBMIs ultimately finds a proper mapping from the user’s intention to control an external device, with sufficient explorations over time. Most of the reported studies show around or over 90% success rates in closed-loop experiments. However, DiGiovanna et al. (2009) reported around 70% success rates in closed-loop experiments. It could be because of the selection of the animal species. Although animals go through behavior training procedures, it is challenging to maintain their engagement during the entire experiment duration. In addition, reaching tasks for the rat require complex associations of all four limbs and entire body movement. In contrast, monkeys can still sit on a chair to conduct reaching tasks by only moving one arm. It is worth noting that relatively lower success rates in open-loop experiments reflect the acquired data are not sufficient to decode complex tasks.

Extracting reward values from the brain is one of the potential advantages of using RL in BMIs, but the majority of the studies fail to address the effect on a choice of a reward modeling method and to explain how the neural signals, extracted for the reward, can be directly communicated to the neural decoder. Few studies have shown possibilities of model reward values in RLBMI based on neural activity from the Nucleus Accumbens (NAcc) (Mahmoudi and Sanchez, 2011; Prins et al., 2017), M1 (Marsh et al., 2015), and mPFC (Shen et al., 2020). However, most of the reported studies solely focus on the performance of the neural decoder by setting reward values based on the experimental setup. Thus, further investigations on the selection of a neural decoder and a reward model are still required.

Furthermore, most studies, which used intracortical signals to validate the neural decoder’s capabilities, showed promising performances and provided detailed setups required for the RLBMI implementations. For example, Bae et al. (2015) explained theoretical properties to guarantee the neural decoder’s convergence. Tarigoppula et al. (2012) provided specific neuron characteristics to boost the neural decoder’s performance. Moreover, Mahmoudi et al. (2013) introduced a learning strategy to adapt a previously learned RL model to a similar task environment. Although these studies are promising, there are many areas for improvement and further exploration. To consider practical implementations of RLBMIs, further investigations of the neural decoders on complex tasks and capabilities for completing sequential tasks are necessary. So far, most studies considered single-step reaching tasks. Although Wang et al. (2017) embedded a 4-target obstacle avoidance task, it was limited to only one subject in an open-loop experiment. One possible approach to address this aspect is to set up sub-goals in RLBMIs (Jurgenson et al., 2019; Paul et al., 2019) that allows a hierarchical sub-goal structure in a task. This will lead to an increased number of steps required to reach a goal and extend the task’s complexity, such as sequential reaching tasks for different goals. Moreover, there is ample room for improving learning speed in RLBMIs. Various transfer learning techniques could be employed to improve the learning rate and to adapt efficiently to changing environments, for instance, when new patterns of neural signals and different types of tasks arise (Tayler and Stone, 2009; Zhu et al., 2020).

In addition, RLBMI literature is mostly comprised of open-loop experiments. This limits their credibility to transfer to real-world scenarios. The number of subjects considered is typically very small, one or two, which is most likely due to the surgical procedures that are required to obtain intracortical signals and behavioral training when recording animals. Therefore, generalization over different subjects has not been investigated yet. In this review, the maximum subject number reported was 20 when EEG was used. Since EEG allows a noninvasive measure of brain activity, it provides an easier and more flexible setup for RLBMIs than the highly invasive intracortical acquisitions. However, it should be noted that EEG brings its own challenges such as degraded signal quality to properly distinguish the different subject’s intentions.

It is well known that due to the nature of the EEG recording process, the spatial resolution is poor, and the signal-to-noise ratio is low (Nicolas-Alonso and Gomez-Gil, 2012). In addition, EEGs are easily contaminated with artifacts, such as movement and electrooculogram (EOG). Therefore, applying additional signal-processing techniques, such as filtering and implementing independent component analysis, become unavoidable (Khosla et al., 2020). In addition, EEG-based BMIs commonly consider imagined motor-imagery targets that are not directly associated with the task itself. That is, due to the challenging separation of neural patterns, experimenters usually assign unrelated imagery to different directions. For instance, Zhang et al. (2019b) implemented an EEG-based BMI where specific instructions to engage a robot to move forward, the subject imagined moving upward, and to turn left, the subject imagined moving downward, and so on. These are unique characteristics of EEG-based BMIs regardless of the employed learning strategy, supervised or reinforcement learning, to tune the parameters of the neural decoder. Studies about neural decoders using RL in EEG-based BMIs are still lacking. There is not enough evidence to provide a conclusive statement about their feasibilities. However, authors want to emphasize its great potential due to its own benefits of the noninvasive recording.

Neural decoders introduced in RLBMIs have shown great potential, and the reported studies encourage further investigations to assess their feasibility. Although RLBMIs are at an early stage to be useful in real-life scenarios, with the aid of advanced RL modeling strategies and signal processing techniques, further investigation might yield more realistic RLBMIs that can be used to assist paralyzed individuals.

## Author Contributions

JB conceived the presented idea, organized the literature, and contributed to the manuscript writing. BG searched the literature, drafted the main table and manuscript, focusing on the search methodology, and neural decoders’ updating rules. WC illustrated diagrams in RLBMIs and summarized fundamental RL backgrounds. All authors contributed to the article and approved the submitted version.

## Conflict of Interest

The authors declare that the research was conducted in the absence of any commercial or financial relationships that could be construed as a potential conflict of interest.

## Publisher’s Note

All claims expressed in this article are solely those of the authors and do not necessarily represent those of their affiliated organizations, or those of the publisher, the editors and the reviewers. Any product that may be evaluated in this article, or claim that may be made by its manufacturer, is not guaranteed or endorsed by the publisher.
